# Transcutaneous carbon dioxide application suppresses the expression of cancer-associated fibroblasts markers in oral squamous cell carcinoma xenograft mouse model

**DOI:** 10.1371/journal.pone.0290357

**Published:** 2023-08-18

**Authors:** Yoshiaki Tadokoro, Daisuke Takeda, Aki Murakami, Nanae Yatagai, Izumi Saito, Satomi Arimoto, Yasumasa Kakei, Masaya Akashi, Takumi Hasegawa

**Affiliations:** Department of Oral and Maxillofacial Surgery, Kobe University Graduate School of Medicine, Kusunoki-cho, Chuo-ku, Kobe; National Hospital Organization Minami Wakayama Medical Center, JAPAN

## Abstract

Oral squamous cell carcinoma (OSCC) is the most common head and neck cancer. Cancer-associated fibroblasts (CAFs) are the main stromal cells in the tumor microenvironment (TME). As CAFs promote tumor progression and hypoxia in the TME, regulating the conversion of normal fibroblasts (NFs) into CAFs is essential for improving the prognosis of patients with OSCC. We have previously reported the antitumor effects of transcutaneous carbon dioxide (CO_2_) application in OSCC. However, the effects of reducing hypoxia in the TME remain unclear. In this study, we investigated whether CO_2_ administration improves the TME by evaluating CAFs marker expression. Human OSCC cells (HSC-3) and normal human dermal fibroblasts (NHDF) were coinjected subcutaneously into the dorsal region of mice. CO_2_ gas was applied twice a week for 3 weeks. The tumors were harvested six times after transcutaneous CO_2_ application. The expression of CAFs markers (α-SMA, FAP, PDPN, and TGF-β) were evaluated by using real-time polymerase chain reaction and immunohistochemical staining. The expression of α-SMA, FAP, PDPN, and TGF-β was significantly increased over time after co-injection. In the CO_2_-treated group, tumor growth was significantly suppressed after treatment initiation. In addition, the mRNA expression of these markers was significantly inhibited. Furthermore, immunohistochemical staining revealed a significant decrease in the protein expression of all CAFs markers in the CO_2_-treated group. We confirmed that transcutaneous CO_2_ application suppressed CAFs marker expression and tumor growth in OSCC xenograft mouse model.

## Introduction

The prevalence of cancer has increased over time, and head and neck cancers (HNCs) are no exception. Oral squamous cell carcinoma (OSCC), of which patients suffer from eating disorders and result in malnutrition, is one of the major HNCs [1, 2]. For treatment planning, surgical resection is the first choice, and radiation therapy and/or chemotherapy are additionally administered to patients with a high risk of recurrence. Various types of chemotherapeutic drugs and radiation therapies are expected to suppress tumor progression with fewer side effects. However, the 5-year survival rate of patients with OSCC remains under 60% and has not improved [3–5]. Local recurrence, metastasis, and drug resistance are the main factors contributing to poor prognosis in OSCC, and the tumor microenvironment (TME) is strongly associated with these factors.

Cancer-associated fibroblasts (CAFs), one of the most dominant spindle-shaped cells in the TME, produce various types of molecular factors and induce tumor cell proliferation, remodel the structure of the extracellular matrix and the metabolic properties of tumor cells, and reprogram the antitumor immune response, metastasis, and acquisition of treatment resistance [6]. Recent studies have revealed that the abundance of CAFs is associated with tumor progression, vascular, lymphatic, and neural invasion, as well as extranodal metastasis. Furthermore, a high presence of CAFs negatively correlates with the overall and disease-free survival of patients with OSCC, suggesting that CAFs can be useful prognostic biomarkers and therapeutic targets [7]. Alpha-smooth muscle actin (α-SMA) and fibroblast activation protein (FAP) are widely used to identify CAFs [8, 9]. Recently, podoplanin (PDPN) and transforming growth factor-β (TGF-β), which stimulate interaction between tumor cells and fibroblasts, have been used as CAF markers [10, 11].

Hypoxia is commonly observed in HNCs owing to cell proliferation and activation in the TME [12]. Hypoxia-inducible factor-1α (HIF-1α) is overexpressed in hypoxic conditions and its expression correlates with tumor cell growth, lymph node metastasis, and poor clinical outcome [13, 14]. In CAFs, the lack of oxygen is known as inducing CAFs reprogramming by stimulating the HIF-1α pathway [15, 16]. Since both CAFs and hypoxia are prominent factors for tumor advancement and clinical outcomes, improving the hypoxic conditions that suppress the expression of CAFs is valuable for patients with OSCC.

Carbon dioxide (CO_2_) therapy is widely known as an effective treatment to improve hypoxia. CO_2_ therapy induces the Bohr effect, an increase in blood flow, and partial pressure of O_2_ under hypoxic conditions. Previous studies have shown that transcutaneous CO_2_ application using CO_2_-absorbing hydrogel promotes hemoglobin-oxygen dissociation and that O_2_ pressure is upregulated in the target area [17, 18]. Takeda et al. reported that this “Artificial Bohr Effect” improved hypoxia and suppressed tumor cell growth in OSCC [19]. Yatagai et al. showed that CO_2_ therapy can improve tumor immunosuppression and chemoresistance [20]. The efficacy of this therapy in tumor cells has been reported; however, no studies have focused on tumor-surrounding cells. Because CAFs are the most common component of the TME that worsen clinical prognosis, suppressing their expression is indispensable. We hypothesized that transcutaneous CO_2_ application downregulates the expression of CAF markers by reducing hypoxia. In this study, we investigated the expression of α-SMA, FAP, PDPN, and TGF-β which indicates CAFs expression, and CO_2_ therapeutic effect on OSCC in a xenograft mouse model.

## Materials and methods

### Cell culture

The oral cell line HSC-3 was purchased from the Health Science Research Resources Bank and established from a metastatic deposit of poorly differentiated SCC of the tongue in the mid-internal jugular lymph node of a 64-year-old man [21]. Normal human dermal fibroblasts (NHDF) were obtained from Lifeline Cell Technology. Both cells were incubated in Eagle’s minimum essential medium supplemented with 10% fetal bovine serum and 1000 units/mL penicillin/streptomycin solution in a 5% CO_2_ atmosphere at 37°C. Trypsin (0.25%) and ethylenediaminetetraacetic acid (0.02%) solutions were used to isolate the cells for subculture [22].

### Xenograft mouse model

We purchased 7-week-old male athymic BALB/cAJcl-nu/nu nude mice from CLEA (Tokyo, Japan). The animal care, welfare, and experiments were performed by trained staff and approved by the Institutional Animal Care and Use Committee and conducted per the Guidelines for Animal Experimentation of the Kobe University Animal Experimentation Regulations (No. P210512). Under anesthesia using isoflurane, HSC-3 cells (1 × 106) were co-injected with NHDF (0.5 × 106) subcutaneously into the dorsal region of each mouse with 500 μL Eagle’s minimum essential medium [23].

### Transcutaneous CO_2_ treatment

We applied a CO_2_ absorption-enhancing hydrogel (CO_2_ hydrogel) to the skin around the tumor site, as previously described [19, 24], which was sealed with a polyethylene bag, and 100% CO_2_ gas was delivered for 20 min. The control animals were similarly treated with air at room temperature instead of CO_2._

### *In Vivo* tumor studies

A total of 25 mice were randomly divided into five groups: 7 d after injection (n = 5), 14 d after injection (n = 5), 21 d after injection (n = 5), CO_2_-treated group (n = 5), and control group (n = 5). Treatment was commenced twice a week for 3 weeks, starting 7 d after the injection. Referring to our previous studies, the endpoint was set at 25 d after injection [19, 20]. Tumor growth and body weight were measured on each day of treatment. Tumor volume was calculated according to the formula, as previously described: V = π/6 × a^2^ × b, where a and b represent the shorter and longer diameters of the tumor [19, 24]. To assess the expression of CAFs over time, tumor tissues were harvested from mice at 7, 14, 21, and 24 h after the end of treatment. Each mouse was weighed and sacrificed under anesthesia before tumor harvest. No animals died before euthanization. Each tumor tissue was divided into two halves; RNA was extracted from one half and paraffin-embedded transverse sections were prepared from the other half.

### Quantitative real-time polymerase chain reaction

HSC-3 cells and NHDF were lysed, and total RNA was extracted using a TRIzol reagent according to the manufacturer’s instructions. Purified RNA was reverse transcribed to cDNA using a high-capacity cDNA Reverse Transcription Kit. The mRNA expression of β-actin, α-SMA, FAP, PDPN, and TGF-β was analyzed by quantitative real-time polymerase chain reaction (qRT-PCR). qRT-PCR was carried out on a StepOne Real-Time PCR System with Power SYBR Green Master Mix per the manufacturer’s instructions. The comparative threshold cycle (2^-△△Ct^) method was used to quantify the relative mRNA expression. β-actin purchased from Invitrogen was used as a reference gene to normalize different samples and designed as previously described [19]. Primers for α-SMA, FAP, and TGF-β were obtained from Sino Biological Inc. PDPN primers were provided by Qiagen.

### Immunohistochemical staining

Specimens were fixed in neutral-buffered formalin and embedded in paraffin. The sections were prepared from paraffin-embedded tissue blocks sliced to 4 μm size. Immunohistochemical staining was performed according to the standard protocol. Briefly, after dewaxing with xylene, rehydration with graded ethanol, and rinsing in deionized water, heat-induced antigen retrieval was performed in a microwave with pH9.0 Tris/EDTA buffer. Next, cool to 37°C or below, 3% hydrogen peroxide was used for blocking endogenous peroxidase activity and were incubated overnight at 4°C with primary antibodies diluted with Can Get Signal Immuno-stain Solution A. Primary antibodies were as follows; rabbit monoclonal α-SMA at a dilution of 1:400, FAP at the dilution of 1:250, PDPN at a dilution of 1:4000, and TGF-β at a dilution of 1:500. An anti-goat IgG polyclonal antibody was added and incubated for 2 h at room temperature. After DAB color development using the peroxidase substrate 3, 3-diaminobenzidine and Hematoxylin contrast staining, the sections were dehydrated, permeabilized, and mounted.

### Immunohistochemical evaluation

The density of CAFs was assessed and classified semi-quantitatively based on the staining intensity and extent of positive staining [6]. Staining intensity was rated on a scale of 0–3 (0 = negative, 1 = weak, 2 = moderate, and 3 = strong). The extent of positive staining was scored as follows: 1, < 20%, 2 = 21–39%, 3 = 40–59%, 4 = 60–79%, and 5, > 80%. The staining intensity and extent scores were multiplied and the Immunoreactive Score (IRS score) was established for each marker. Theoretically, IRS could range from 0 to 15, and the score over 10 was determined as strongly positive (+ + +), 7 to 9 moderate positive (+ +), 4 to 6 weak positive (+), and 0 to 3 negative (-). Six high-power fields were randomly selected and scored by two investigators (Y.T. and T.H.). For PDPN, the fibroblast area was selected and the positive staining rate was evaluated. Before data analysis, cases with interobserver discrepancies were re-evaluated until a consensus was reached.

### Statistical analysis

Data are presented as means and standard deviations. Statistical analyses of the data were performed using Mann–Whitney U or Steel-Dwass tests. The statistical significance was level set at p < 0.05.

## Results

### The expression of CAFs markers increases over time

**Fig 1:**
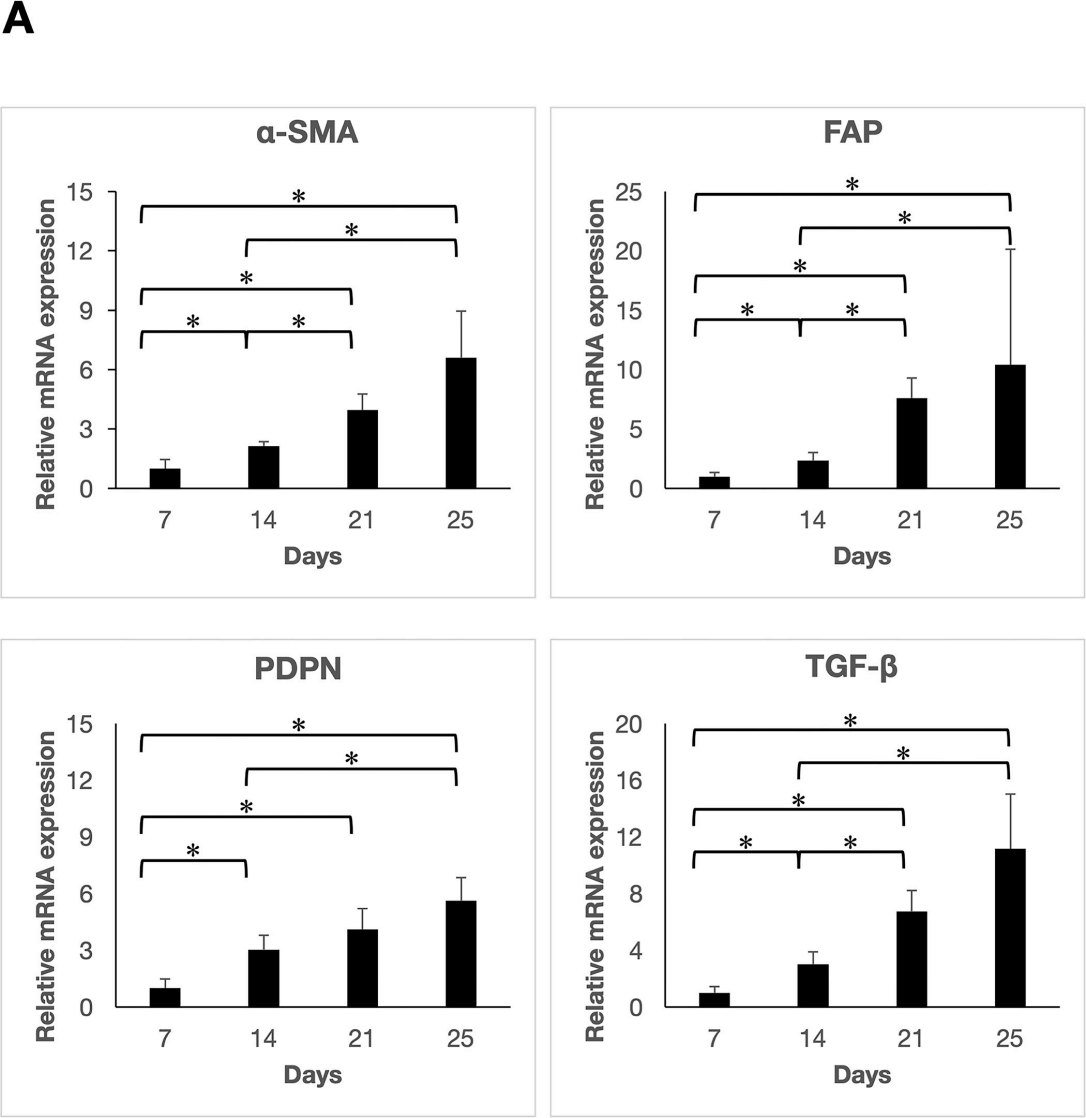
The evaluation of the CAFs marker on each treatment day. (A) qRT-PCR analysis. The mRNA expression was evaluated weekly and at the endpoint. The expression of all the markers increased significantly over time *p < 0.05). (B) Immunohistochemical staining. All images were recorded at 200×magnification. (C) Immunohistochemical Evaluation. The IRS scores for all markers significantly increased over time *p < 0.05).

To investigate the expression of CAFs markers, we first performed qRT-PCR and immunohistochemical staining on days 7, 14, 21, and 25 after co-injection. The mRNA expression of α-SMA, FAP, PDPN, and TGF-β was confirmed on Day 7 and significantly increased over time (Fig 1A). In immunohistochemical analysis, all markers were negative on Day7, however, the IRS score showed a statistically significant increase until the mice were sacrificed (Fig 1B and 1C). These results suggest that CAFs marker expression continued to increase over time in the xenograft mouse model in which HSC-3 cells and NHDF were co-injected.

### Transcutaneous CO_2_ application suppresses tumor growth

**Fig 2:**
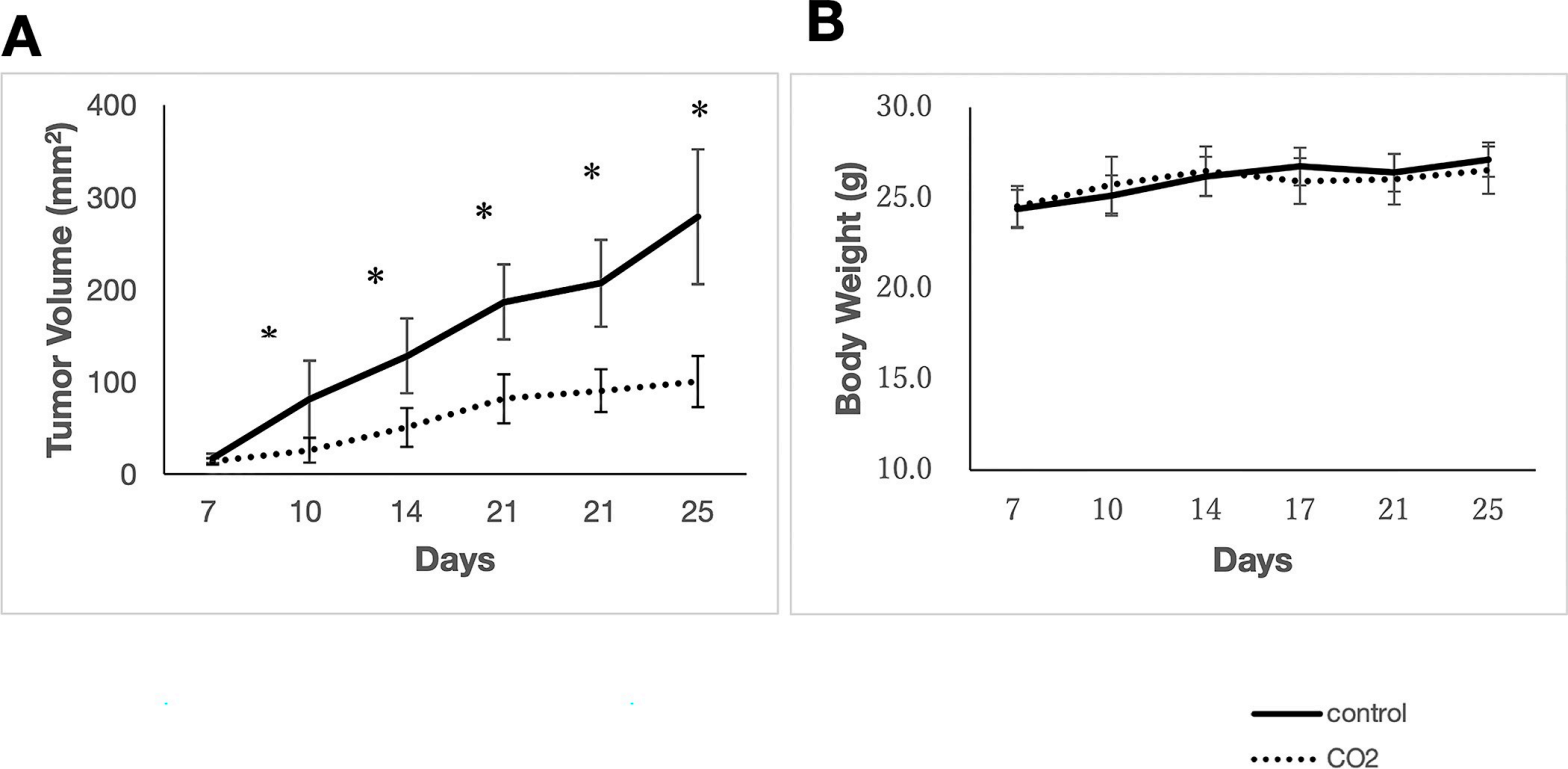
Tumor volume and body weight of mice in control and CO_2_-treated groups. (A) Tumor volume. Tumor growth was significantly suppressed at each time point after CO_2_ administration. *p < 0.05 (B) Body weight of mice. No significant differences were observed.

We observed the effect of transcutaneous CO_2_ application on tumor growth and found significant suppression in the CO_2_-treated group after treatment initiation (p < 0.05; Fig 2A). Furthermore, tumor growth was significantly suppressed on day 25, at the end of the treatment; control group (278.9 ± 73.1 mm^3^) vs CO_2_-treated group (100.3 ±27.6 mm^3^, p < 0.05, Fig 2A). In addition, CO_2_-treated group weighed slightly less than the control group at 14 d after injection. However, no significant differences were observed between the groups (Fig 2B).

### Transcutaneous CO_2_ application reduces intratumor hypoxia and significantly suppresses the expression of CAFs markers

**Fig 3:**
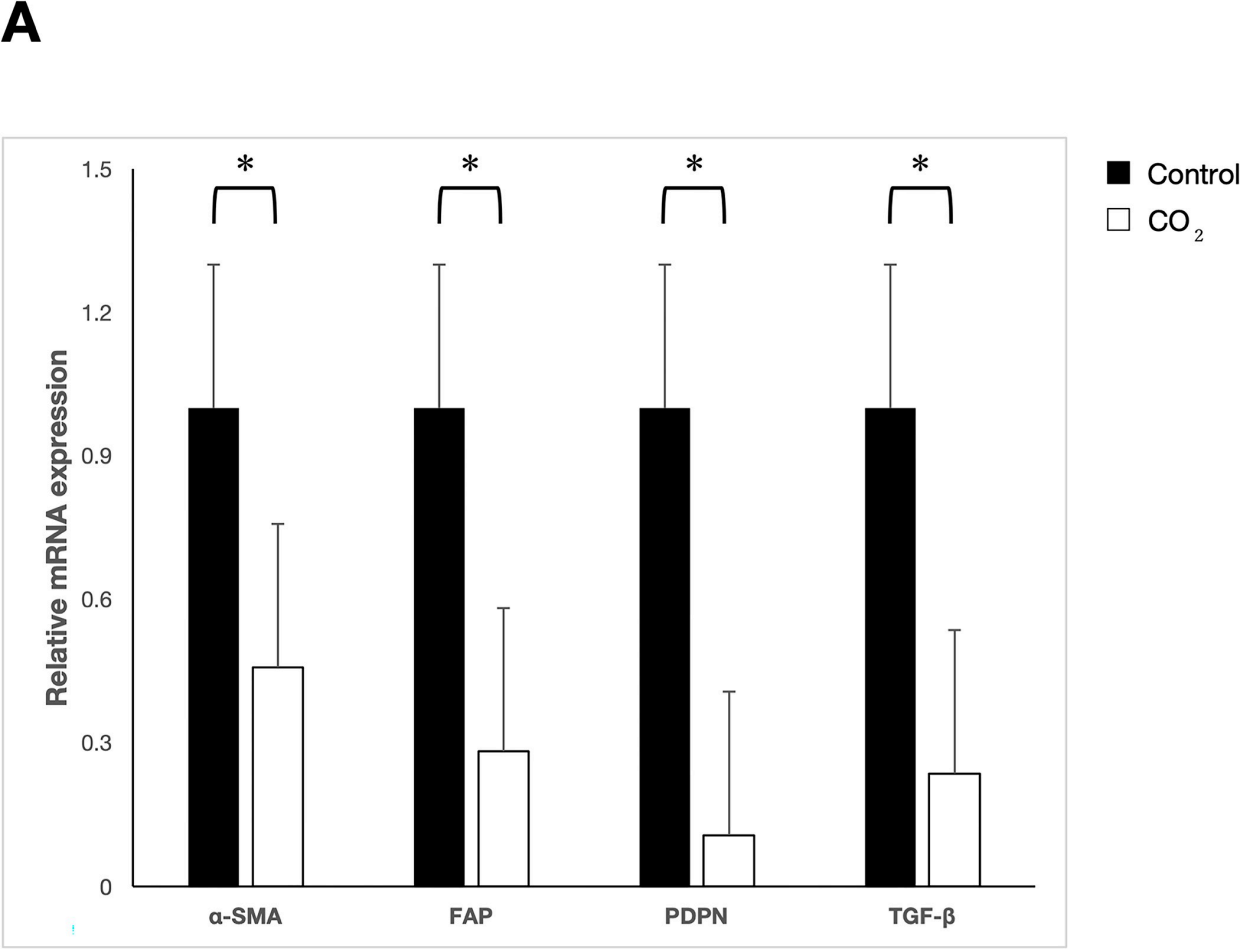
The effect of transcutaneous CO_2_ treatment. (A) qRT-PCR analysis. The expression of each marker was evaluated on Day 25. Compared with the control group, the expression of α-SMA, FAP, PDPN, and TGF-β was significantly suppressed in the CO_2_-treated group. *p < 0.05. (B) Immunohistochemical staining (magnification: 200×). (C) Immunohistochemical evaluation. The IRS score of all markers was significantly suppressed in the CO_2_-treated group. *p < 0.05.

To evaluate the effect of CO_2_ treatment on CAFs marker expression, we performed qRT-PCR and determined the mRNA expression in implanted tumors. In the CO_2_-treated group, the mRNA expression of α-SMA, FAP, PDPN, and TGF-β was significantly inhibited compared with the control group (p < 0.05, Fig 3A). Immunohistochemical analysis showed that these markers were significantly suppressed in the CO_2_-treated group (p < 0.05; Fig 3B and 3C). Together, these results indicated that transcutaneous CO_2_ application significantly suppressed the expression of CAF markers.

## Discussion

Despite recent advances in the treatment of malignancies, the mortality rate in patients with OSCC remains high [3]. Uncontrolled tumor progression is attributed not only to genetic mutations but also to various growth factors secreted by adjacent stromal cells and signaling pathways activated by cell-cell interactions [25]. CAFs, which are associated with common modes of tumor spread and metastasis, including perineural invasion, vascular invasion, and lymph node metastasis, play a pivotal role in the TME.

CAFs are heterogeneous populations with diverse origins, including NFs, bone marrow-derived mesenchymal stem cells, hematopoietic stem cells, epithelial cells, and vascular endothelial cells [26]. Their heterogeneous nature and high plasticity make it difficult to establish a unique marker for CAFs. Several markers such as α-SMA and FAP are commonly combined to identify CAFs [8, 9, 26]. α-SMA is an intracellular actin stress fiber formed during fibroblast activation, which is strongly associated with the formation of cell motility, structure, and integrity. Because the number of myofibroblasts significantly increases in the TME, α-SMA is regarded as one of the valid markers for identifying CAFs populations [27, 28]. FAP, a type II integral membrane protein, is highly expressed in fibroblasts and pericytes and is active in wound healing, fibrosis, and extracellular matrix degradation [29, 30]. Many studies have used FAP as a CAFs marker, as it is strongly expressed and upregulated in the epithelial tumor stroma [8, 31].

Although not specific for NFs, PDPN is overexpressed and correlates with α-SMA expression in CAFs populations and has recently emerged as a CAFs marker [10, 32, 33]. PDPN is a mucin-like transmembrane glycoprotein that is mainly located on the invasive fronts of OSCC, which is consistent with its role in promoting invasion. It is widely recognized as a lymphatic endothelial marker [34]. Li et al. described that not only PDPN overexpression in OSCC cells induces NFs activation but also CAFs induced PDPN expression via the TGF-β signaling pathway [10]. TGF-β, a secreted multifunctional cytokine that plays a key role in tumor occurrence, development, and metastasis, is also responsible for NFs activation, which means CAFs expression [35, 36]. A recent study revealed that TGF-β promotes the reprogramming of NFs into CAFs in patients with HNCs [11]. For these reasons, TGF-β was selected as an indispensable marker to identify CAFs expression in this study.

High CAFs density is associated with poor patient prognosis and local recurrence [6, 37, 38]. Kreppel et al. reported that the degree of PDPN expression correlates with lymphoid metastasis and 5-year disease-specific survival rates of patients [39]. TGF-β not only promotes cancer cell–stroma interaction and CAFs-mediated changes in the extracellular matrix but also limits cetuximab efficacy [40, 41]. Hence, suppression of these markers is crucial for improving clinical outcomes.

Hypoxia is one of the most prominent features of tumor development and progression [42]. In solid tumors, cell proliferation causes an insufficient blood supply and results in excess oxygen and nutrient supply, leading to hypoxia in the TME [12]. Oxygen deficiency is associated with the invasiveness and metastatic ability of tumor cells, and leads to poor prognosis in patients with OSCC [13, 14]. Lack of oxygen has recently been considered one of the factors that convert NHDFs into CAFs. In the hypoxic region, the tumor cell secretes TGF-β and stimulates the HIF-1α pathway. This pathway is implicated in glycolysis which induces NFs/CAFs conversion [15, 16, 43]. These CAFs produce ECM, which differs from normoxic conditions and promotes tumor cell migration.

As mentioned above, CAFs strongly correlate with hypoxic conditions and are not only reprogrammed but also stimulate tumor progression. To suppress CAFs expression by reducing the hypoxic environment, we considered transcutaneous CO_2_ application as an effective treatment. CO_2_ administration is known to locally improve microcirculation and partial oxygen pressure. Transcutaneous CO_2_ can increase O_2_ pressure by releasing O_2_ from red blood cells, that is, through the Artificial Bohr Effect” [17, 44]. We have previously demonstrated that this Bohr effect decreases the expression of HIF-α, which is associated with tumor progression, lymph node metastasis, acquisition of chemotherapy and radiation therapy, and clinical outcome by improving hypoxia condition. This application also induces tumor cell apoptosis and down-regulates tumor growth in OSCC [19, 24, 45]. More recently, we found the possibility of suppressing tumor immunity by combining this treatment with chemotherapy, showing that CO_2_ treatment can be used as adjuvant therapy [20]. Although many previous studies have focused solely on tumor cells, no study has reported the therapeutic effects of targeting the TME. We are the first to find that this application influences CAFs expression by improving hypoxia in the TME and downregulating the crosstalk between tumor cells and stromal cells.

Surgical resection is the standard treatment for OSCC. However, oral dysfunction and facial deformities remain serious problems for patients. Because CAFs are strongly associated with tumor invasion, this study indicates that transcutaneous CO_2_ application has the potential to localize tumors. This indicates that CO_2_ therapy can be applied preoperatively to reduce the resection area and preserve function and morphology in patients with OSCC. Compared with other preoperative therapies such as induction chemotherapy, this application has fewer side effects in controlling tumor growth and invasion. This study had several limitations. First, it is not known whether transplanted NHDFs have differentiated into CAFs, because CAFs are heterogeneous populations with diverse origins. The mechanism of NFs/CAFs conversion has not been fully revealed. Secondly, although CO_2_ therapy is simple and inexpensive, several problems remain in its clinical application. Given the difficulty in administering CO_2_, the application of this therapy can be easily limited by tumor sites, such as the oropharynx or the posterior part of the tongue. In addition, patients with a vomiting reflex or limited mouth opening have difficulty receiving CO_2_. To overcome these problems, we have developed a CO_2_ paste that does not produce gaseous CO_2_ and is efficiently absorbed by the skin [46]. In the future, we hope to clarify the efficacy of this paste in inhibiting HNCs progression.

## Conclusion

We investigated the effects of CO_2_ application on CAFs marker expression of the poorly differentiated OSCC neck metastasis cell and NHDF co-injected in a xenograft mouse model. We first reported that transcutaneous CO_2_ application suppressed CAFs marker expression and tumor growth in a xenograft mouse model.

## Supporting information

S1 Fig Minimal dataset for quantitative real-time polymerase chain reaction analysis. (XLSX)

S2 Fig Minimal dataset for immunohistochemical evaluation, IRS score. (XLSX)

S3 Fig Minimal dataset for tumor volume and body weight. (XLSX)
