# Correction: Transcutaneous carbon dioxide application suppresses the expression of cancer-associated fibroblasts markers in oral squamous cell carcinoma xenograft mouse model

**DOI:** 10.1371/journal.pone.0307334

**Published:** 2024-07-11

**Authors:** The PLOS ONE Staff

Fig 1B and 1C, “The evaluation of the CAFs marker on each treatment day.” does not appear. Please view Fig 1 here.

**Fig 1:**
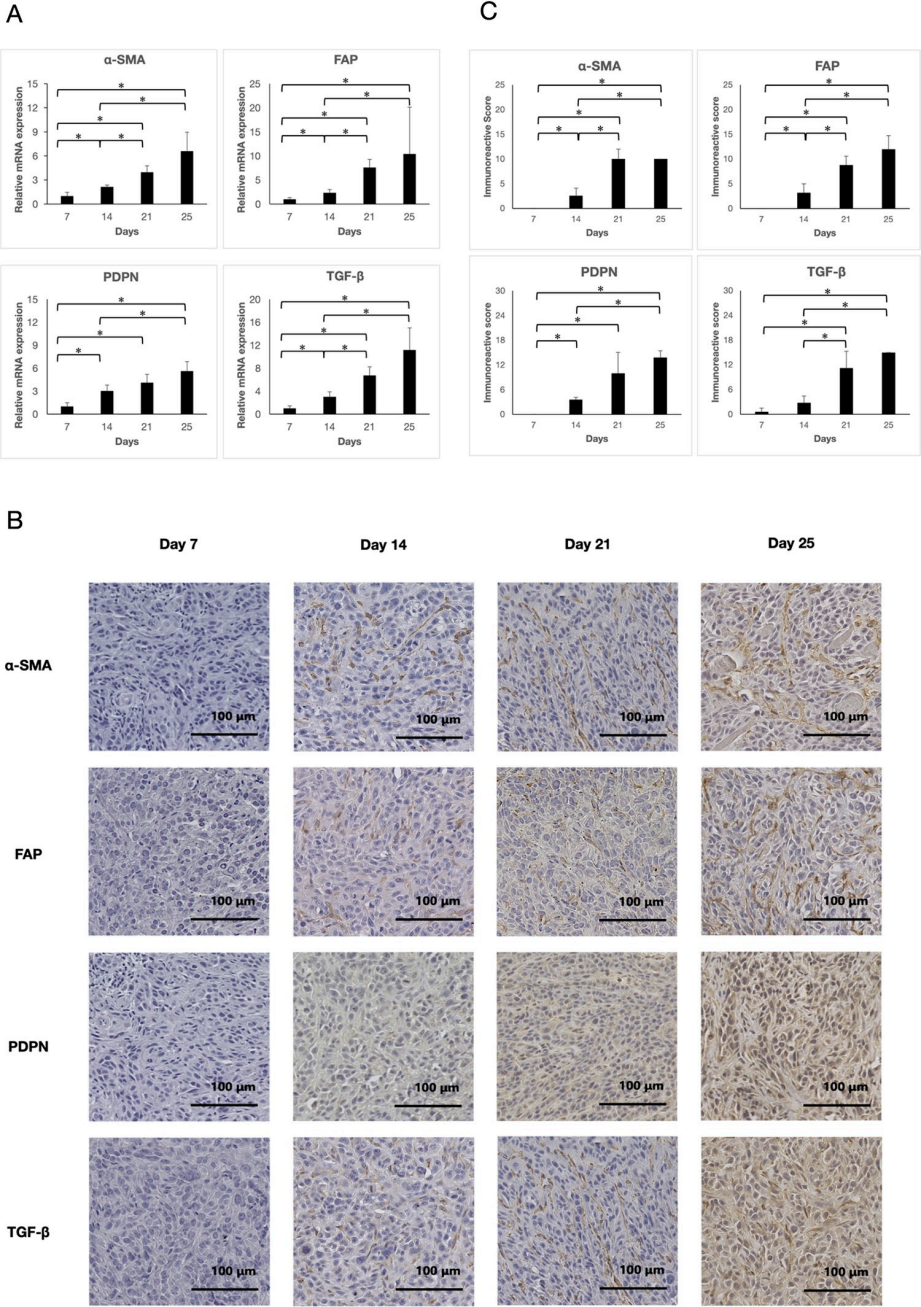
The evaluation of the CAFs marker on each treatment day. (A) qRT-PCR analysis. The mRNA expression was evaluated weekly and at the endpoint. The expression of all the markers increased significantly over time *p < 0.05). (B) Immunohistochemical staining. All images were recorded at 200×magnification. (C) Immunohistochemical Evaluation. The IRS scores for all markers significantly increased over time *p < 0.05).

Fig 3B and 3C, “The effect of transcutaneous CO2 treatment.” does not appear. Please view Fig 3 here.

**Fig 3:**
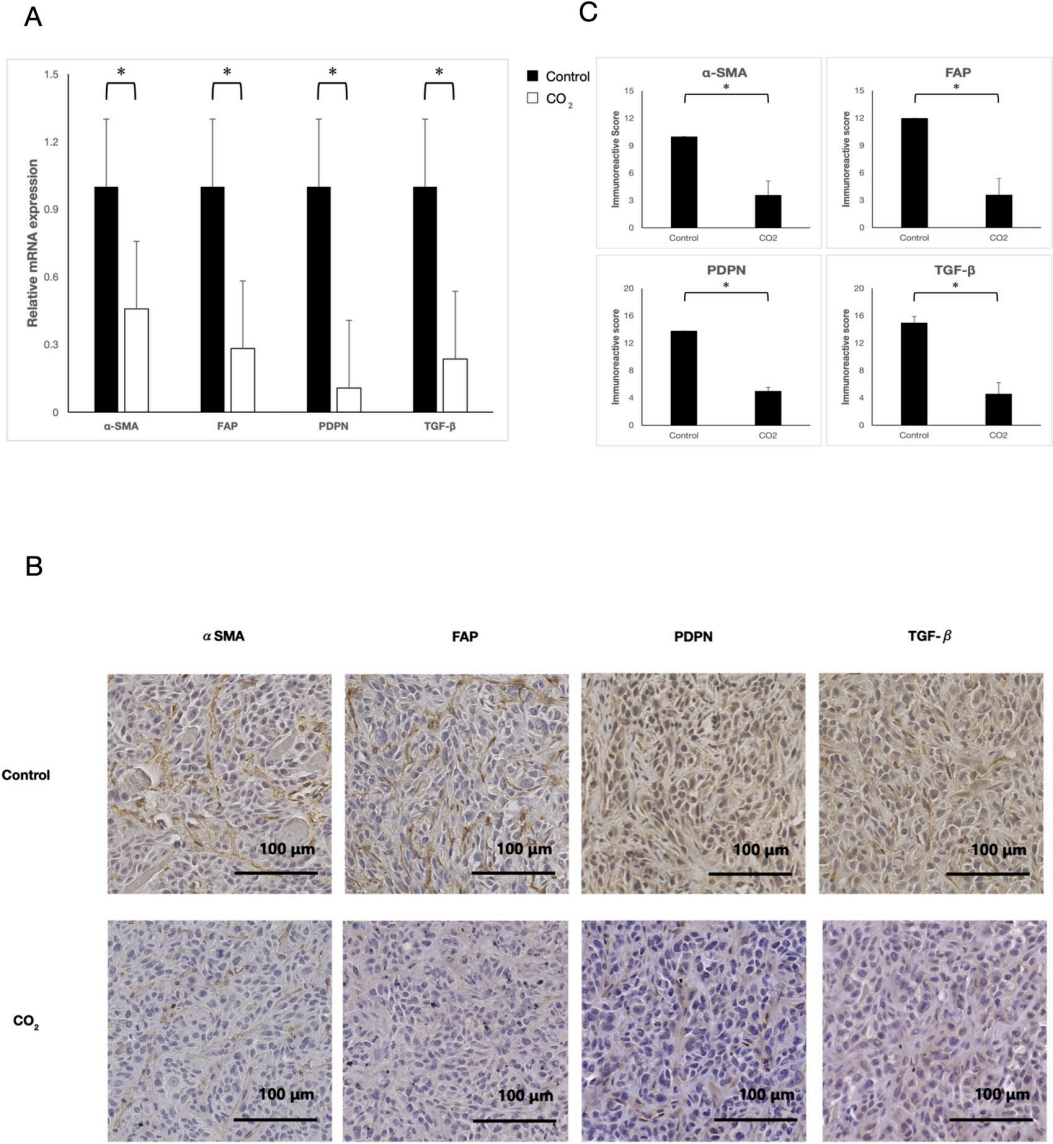
The effect of transcutaneous CO2 treatment. (A) qRT-PCR analysis. The expression of each marker was evaluated on Day 25. Compared with the control group, the expression of α-SMA, FAP, PDPN, and TGF-β was significantly suppressed in the CO2-treated group. *p < 0.05. (B) Immunohistochemical staining (magnification: 200×). (C) Immunohistochemical evaluation. The IRS score of all markers was significantly suppressed in the CO2-treated group. *p < 0.05.

The publisher apologizes for the errors.
